# Aldo-Keto Reductases in the Eye

**DOI:** 10.1155/2010/521204

**Published:** 2010-06-13

**Authors:** Shun Ping Huang, Suryanarayana Palla, Philip Ruzycki, Ross Arjun Varma, Theresa Harter, G. Bhanuprakesh Reddy, J. Mark Petrash

**Affiliations:** ^1^Department of Ophthalmology & Visual Sciences, Washington University School of Medicine, St. Louis, MO 63110, USA; ^2^Program in Genetics, Molecular, and Cell Biology, University of Southern California School of Medicine, Los Angeles, CA 90033, USA; ^3^Biochemistry Division, National Institute of Nutrition, Hyderabad-500 604, India; ^4^Department of Ophthalmology, Rocky Mountain Lions Eye Institute, University of Colorado Denver, Aurora, CO 80111, USA

## Abstract

Aldose reductase (AKR1B1) is an NADPH-dependent aldo-keto reductase best known as the rate-limiting enzyme of the polyol pathway. Accelerated glucose metabolism through this pathway has been implicated in diabetic cataract and retinopathy. Some human tissues contain AKR1B1 as well as AKR1B10, a closely related member of the aldo-keto reductase gene superfamily. This opens the possibility that AKR1B10 may also contribute to diabetic complications. The goal of the current study was to characterize the expression profiles of AKR1B1 and AKR1B10 in the human eye. Using quantitative reverse transcriptase-PCR and immunohistochemical staining, we observed expression of both AKR genes in cornea, iris, ciliary body, lens, and retina. Expression of AKR1B1 was the highest in lens and retina, whereas AKR1B10 was the highest in cornea. Lenses from transgenic mice designed for overexpression of AKR1B10 were not significantly different from nontransgenic controls, although a significant number developed a focal defect in the anterior lens epithelium following 6 months of experimentally induced diabetes. However, lenses from AKR1B10 mice remained largely transparent following longterm diabetes. These results indicate that AKR1B1 and AKR1B10 may have different functional properties in the lens and suggest that AKR1B10 does not contribute to the pathogenesis of diabetic cataract in humans.

## 1. Introduction

Diabetes mellitus is recognized as a leading cause of new cases of blindness among Americans between the ages of 20 and 74. At least 5,000 new cases of legalblindness result each year from diabetic retinopathy alone [1]. The incidence of cataract is also much higher in diabetic than in nondiabetic individuals [2]. Many theories have been advanced to explain the pathogenesis of diabetic eye disease. These include excess formation of advanced glycation end-products [3], activation of PKC isoforms [4], activation of the polyol pathway [5], and excessive oxidative stress [6]. Considerable evidence points to excess conversion of glucose to sorbitol, mediated by aldose reductase (AKR1B1),as a key factor in diabetic cataract formation. AKR1B1-mediated polyol accumulation causes osmotic imbalances that lead to fiber cell swelling, liquefaction, and eventually cataract [5]. Compelling evidence to support this hypothesis came from Lee and coworkers, who created a transgenic mouse model that expressed high levels of AKR1B1 in lens fiber cells [7]. These mice developed cataracts following diabetes induction, demonstrating an essential role for AKR1B1 in mediating high glucose-dependent cataract formation. 

The role of AKR1B1 during euglycemia is still unclear. The aldo-keto reductase (AKR) gene superfamily includes several enzymes and proteins with similar structures and/or enzymatic activities. The AKR1B subfamily contains two genes that are expressed at relatively high levels in human tissues. AKR1B1, which is equivalent to aldose reductase, is expressed in many tissues throughout the body. AKR1B10, which has been given the trivial names human small intestine reductase (HSIR) and AKR1B1-like protein 1 (ARL-1), is also expressed in many tissues [8, 9]. Based on a blot analysis of multiple tissue RNAs, gene transcript levels of AKR1B10 closely parallel those of AKR1B1 [8]. The broad catalytic similarities between AKR1B1 and AKR1B10 make it difficult to map the distribution of these proteins in human tissues using enzyme activity assays. The enzymes utilize an overlapping array of substrates, and many so-called aldose reductase inhibitors effectively block both AKR1B1 and AKR1B10 [10]. Therefore, studies conducted over 2 decades ago to demonstrate expression of AKR1B1 in tissues of the human eye may have lacked sufficient specificity to distinguish between these two closely related gene products [11, 12]. In the current study, we have reexamined the expression pattern of these enzymes, taking into account the possibility that AKR1B10 may contribute to the aldo-keto reductase profile of ocular tissues and thus may participate in the pathogenesis of diabetic eye disease. The current study also addressed the question of whether AKR1B10 contributes to the onset and progression of cataracts in a mouse model of diabetes.

## 2. Materials and Methods

### 2.1. Human Eyes and Specimens

Human postmortem eyes were obtained from certified eye banks through the National Disease Research Interchange. The time interval between death to enucleation (<8 hours) and then to fixation (usually 8–12 hours) was rigorously controlled. Once received in the laboratory, tissues were handled under RNAse-free conditions. The cornea, iris, ciliary body, lens, and retinas were carefully dissected and used to prepare protein lysates.

### 2.2. Quantitative Real-Time PCR (qRT-PCR)

Total RNA was extracted from human ocular tissues using an RNase kit (Qiagen). After digesting genomic DNA using DNase I (Roche), cDNA was synthesized from 1 *μ*g total RNA using Retroscript Kit (Ambion) in 20 *μ*L volume. Quantitative real-time PCR for AKR1B1 and AKR1B10 were done using an iCycler iQ Detection System (Bio-Rad, Hercules, CA). Reaction mixtures contained iQ SYBR Green Supermix (Bio-Rad) and primers 5′for-CCCAAAGATGATAAAGGTAATGCCATCGGT-3′ and 5′rev-CGATCTGGAAGTGGCTGAAATTGGAGA-3′ for AKR1B10, 5′for-TGAGTGCCACCCATATCTCA-3′ and 5′rev-TGTCACAGACTTGGGGATCA-3′ for AKR1B1, or 5′for-AGAAGGAGATCACTGCCCTGGCACC and 5′rev-CCTGCTTGCTGATCCACATCTGCTG for *β*-actin. PCR condition was 1 cycle of 95°C for 3 minutes followed by 40 cycles at 95°C for 20 seconds, 65°C for 30 seconds, and 72°C for 30 seconds. All the samples were run in triplicate, and the results were averaged. Specific amplification of AKR1B1, AKR1B10, and *β*-actin (244 bp for AKR1B1, 133 bp for AKR1B10 and 162 bp for *β*-actin) was confirmed by gel electrophoresis and melting curve analysis after PCR. In order to compare expression patterns among tissues, relative quantification of gene expression was performed using the standard curve method. The quantification data of AKR1B1 and AKR1B10 was indicated as a relative ratio of its signal to that of *β*-actin to normalize the starting amount of template cDNA. 

### 2.3. Western Blot Analysis

Proteins were isolated from human ocular tissues by treatment in lysis buffer (10 mM Tris, pH 7.4, 150 mM NaCl, 1% Triton X-100, 1 mM EDTA, pH 8.0, 1 mM EGTA, pH 7.0, 0.2 mM sodium orthovanadate, 1 mM PMSF, 0.5% NP-40) with freshly added aprotinin to a final concentration of 5 *μ*g/mL. Protein concentration was determined with the bicinchoninic acid methods, using BSA as standard (Micro BCA Protein Assay Kit; Pierce, Rockford, IL). Equivalent amounts of protein (40 *μ*g) from total cell lysates or tissue lysates were boiled in Nupage LDS sample buffer (Invitrogen) for 5 minutes and analyzed by 10% SDS-PAGE. Separated proteins were transferred to Hybond-P PVDF membrane and were blocked with TBS-0.1% Tween-20 containing 5% nonfat milk for overnight. Membranes were incubated with antibodies for AKR1B1 (1 : 3000) or AKR1B10 (1 : 3000 dilution), probed with horseradish peroxidase-conjugated antirabbit secondary antibody (1 : 8000) for 2 hours, and washed. Immune complexes were visualized with the ECL plus system and scanned on a STORM 860 phosphorimager. Membranes were washed and reprobed with anti-*β*-actin antibody. Recombinant AKR1B1 or AKR1B10 proteins were used as size standards. 

### 2.4. Antibody Preparation

Antibodies to AKR1B1 and AKR1B10 were prepared through a commercial service (Bethyl Laboratories, Montgomery, TX). Antibodies to human AKR1B1 were made by immunizing rabbits with recombinant human AKR1B1, purified as described previously [13]. Antibodies to AKR1B10 were prepared using synthetic peptides derived from AKR1B10 encompassing residues 120 to 134 (CDDLFPKDDKGNAIGG). In both cases, antibodies were purified by column chromatography using the immunogen bound to a solid phase support as the affinity ligand. Antibody specificity was verified using purified recombinant AKR1B1 and AKR1B10 in a western blotting format (data not shown).

### 2.5. Immunohistochemistry and Immunofluorescence

Immunohistochemical analysis for AKR1B1 and AKR1B10 was done with the formalin-fixed, paraffin-embedded tissue. The sections were deparaffinized in xylene, incubated for 30 minutes in methanol containing 3% H_2_O_2_ to inhibit endogenous peroxidase activity, rehydrated through a series of graded alcohols, and stained for AKR1B1 or AKR1B10 via the immunoperoxidase technique. The tissue was covered with 20% inactivated normal donkey serum in Tris-buffered saline, pH 7.6, incubated for 30 minutes at room temperature, and blotted and incubated overnight with a 1 : 500 dilution of AKR1B1 antiserum or a 1 : 500 dilution of rabbit anti-AKR1B10 peptide antibody overnight at 4°C. Goat antirabbit antibody was used as a secondary antibody after 500-fold dilution. Preimmune serum was used on sections serving as negative controls. Immunostaining was visualized using diaminobenzidine tetrahydrochloride (DAB), a horseradish peroxidase system (Vector Laboratories, Burlingame, CA). Sections were counterstained with hematoxylin. Areas of positive reactivity are stained brown.

### 2.6. Transgenic Mice

Transgenic mice on C57BL6 background were produced for lens-enriched expression of AKR1B10. The transgene construct was prepared by ligating the hybrid *α*/*δ*-crystallin promoter [14] to a complete cDNA sequence encoding AKR1B10. Further details on the preparation and characterization of five independent founder lines of AKR1B10 transgenic mice will be presented elsewhere. Animals derived from founder line PAR30 used in this study were maintained by outbreeding to the C57BL6 strain obtained from Jackson Laboratories (Bar Harbor, ME). 

### 2.7. Diabetes Induction

Experimental diabetes was induced in transgenic and nontransgenic control mice by treatment with a low-dose regimen of streptozotocin as described [15]. Fasting blood sugars were measured starting 2 weeks after the final streptozotocin treatment and monthly after hyperglycemia was established. For blood sugar measurements, animals were fasted for 6 hours prior to collection of a drop of blood from the saphenous vein. Glucose levels were measured immediately using a glucometer (AlphaTRAK, Abbot Laboratories, Chicago, IL). Animals were included in the study if fasting glucose levels were 250–350 mg/dL. 

## 3. Results

### 3.1. Aldo-Keto Reductases in the Human Eye

We used a quantitative real-time PCR-based assay (qRT-PCR) for measuring the expression profiles of the AKR1B1 and AKR1B10 genes in human eye tissues. The RT-PCR method was chosen because it provided the specificity necessary to distinguish between AKR1B1 and AKR1B10 gene transcripts, unlike the case with standard nucleic acid hybridization methods such as Northern blotting.

**Figure 1:**
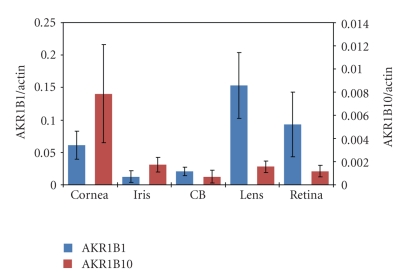
Expression of AKR1B1 and AKR1B10 in human eye tissues. Gene transcript levels were measured by quantitative real-time PCR as described in Section 2. Data are mean ± SD among 5 nondiabetic male donors aged 65. ± 9.2 years. Data for AKR gene transcripts levels are normalized to RT-PCR for *β*-actin.

The expression profiles for AKR1B1 and AKR1B10 mRNA levels were measured in cornea, iris, ciliary body, lens, and retina. Data on the apparent abundance of gene-specific transcripts were computed relative to *β*-actin and are shown in Figure 1. Transcripts derived from the AKR1B1 gene are present in all tissues examined, and are the highest in lens followed by retina and cornea. In the case of AKR1B10 gene transcripts, the highest transcript levels are found in cornea, with substantially lower levels found in iris, ciliary body, lens, and retina. 

**Figure 2:**
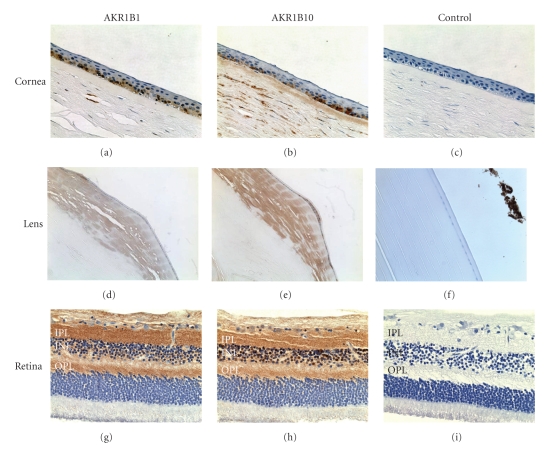
Immunostaining for AKR1B1 and AKR1B10 in human eye tissues. A donor eye (77-year-old male) was treated with antibodies to AKR1B1 and AKR1B10 or preimmune control serum. Immune complexes were visualized by treatment with a horseradish peroxidase-conjugated secondary antibody and signal developed using diaminobenzidine tetrahydrochloride (DAB) to give a brown color. Tissues examined include cornea (a)–(c), lens (d)–(f), and retina (g)–(i). Immunostaining was particularly strong in the inner plexiform (IPL) and outer plexiform (OPL) layers. The inner nuclear layer is shown (INL).

To examine the distribution of the AKR1B1 and AKR1B10 at the protein level, we carried out immunohistochemical staining of paraffin sections produced from human eyes using affinity purified antibodies prepared as described in Section 2. The results are shown in Figure 2 and can be summarized as follows.

**Cornea**

Both AKR1B10 and AKR1B1 are expressed in the corneal epithelium. Staining appeared to be stronger in the basal as compared to the superficial cell layer. Intense staining for AKR1B10 was observed in the corneal stroma whereas AKR1B1 staining was relatively limited in this region.

**Lens**

Staining for AKR1B1 and AKR1B10 was observed in the lens epithelium and fiber cells located in the superficial cortex. The staining was the greatest at the equator region and diminished in cells located deeper in the cortex and nucleus.

**Retina**

AKR1B1 and AKR1B10 stained heavily in cell nuclei in the inner nuclear layer as well as in some ganglion cells, especially near the perinuclear cytoplasm and inner limiting membrane. Intense staining was also observed in the inner and outer plexiform layers. No significant immunohistochemical staining of AKR1B10 could be observed around the retinal vessels. In all cases, no staining positivity was observed when the primary antibody was omitted.

**Transgenic Mice**

**Figure 3:**
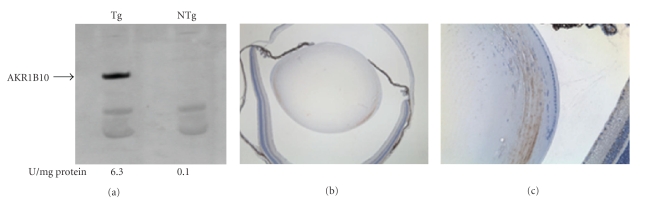
AKR1B10 expression in the transgenic lens. (a) Western blot demonstrating expression of AKR1B10 in lens of transgenic (Tg) mice; the characteristic band was not observed in lenses from nontransgenic (NTg) controls. Aldo-keto reductase enzyme activity in lenses is shown below each lane. (b) and (c) Immunohistochemical stain for AKR1B10 expression in the transgenic lens.

Based on results from RT-PCR and immunostaining experiments, it appears that both AKR1B1 and AKR1B10 are expressed in the human lens. To assess whether high levels of AKR1B10 can predispose the lens toward diabetic cataract, we produced transgenic mice designed for overexpression of the enzyme in the lens. As shown in Figure 3, AKR1B10 was readily detected by western blotting of lens homogenates from transgenic animals but was absent in nontransgenic control lenses. Immunohistochemical staining showed intense positivity in the outer cortical fiber cells of transgenic animals and no detectable staining of nontransgenic controls (Figure 3). Thus, expression of AKR1B10 in the transgenic lens had a similar regional distribution as endogenous AKR1B10 in the human lens. On a gross level, overexpression of AKR1B10 did not have a measureable impact on lens development, as the wet weight and appearance of transgenic lenses was not significantly different from nontransgenic controls (data not shown).

**Figure 4:**
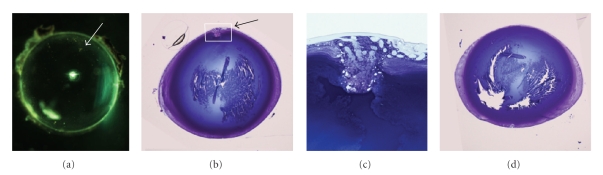
Lens defect in AKR1B10 lens after long-term diabetes. (a) Brightfield microscopy of transgenic lens demonstrating light scattering defect (arrow). (b) AKR1B10 transgenic lens showing defect at the anterior aspect of the lens (arrow). (c) Magnification of the boxed area from panel (b). (d) Lens from nontransgenic control with equivalent duration of diabetes. Panels (b)–(d) are from toluidine blue-stained lenses.

We induced experimental diabetes in our transgenic mice to determine if over-expression of AKR1B10 influences the susceptibility of the mouse lens to cataracts. Both transgenic and nontransgenic animals with and without experimental diabetes were monitored for up to six months for the appearance of lens opacities. In all cases, the lenses remained essentially clear and developed only minor focal areas of light scattering in the lens nucleus, typical of the normal aging mouse lens [16]. A refractive abnormality localized on the anterior epithelium was observed in 50% (3 of 6) of the AKR1B10 mice with diabetes. This defect gave rise to light scattering when viewed through a slit lamp ophthalmoscope or after dissection and brightfield illumination (Figure 4). Histological examination showed that this abnormality was associated with a localized disorganization of epithelial cells, formation of large vacuoles, and disrupted contact between epithelial cells and the lens capsule. This defect was not observed in age-matched nontransgenic controls with equivalent duration of experimental diabetes (*n* = 4) or in nondiabetic transgenic controls (*n* > 6). The epithelial defect we observed is fundamentally different from cortical opacities that characterize the majority of diabetic cataracts.

## 4. Discussion

Cataract formation is a major complication of diabetes. Osmotic stress to lens fiber cells resulting from excessive production and/or accumulation of sorbitol has been proposed as a mechanism leading to diabetic cataracts in humans. Varma and coworkers previously demonstrated a strong correlation between the abundance of polyol pathway metabolites sorbitol and fructose and blood glucose levels in cataracts extracted from diabetic patients [17]. Our gene expression profiling of AKR1B1 and AKR1B10 using gene-specific RT-PCR clearly demonstrated that both of these aldo-keto reductases are expressed not only in lens but also in cornea, retina, and ciliary body. This raised the possibility that diabetes-induced cataract and retinopathy, as well as increased risk for glaucoma and corneal abnormalities, may develop through multiple AKR-linked mechanisms. 

We employed a genetic strategy to determine if AKR1B10 contributes to the pathogenesis of diabetic cataract in a mouse model. Mouse lenses contain insignificant levels of AKR1B3, the mouse ortholog of human AKR1B1. Other members of the AKR1B subfamily, such as AKR1B7 (major vas deferens protein, MVDP; 18) and AKR1B8 (fibroblast growth factor-induced protein 1; FR-1; 19), are virtually undetectable in the mouse lens. Previous studies by Lee et al. demonstrated that transgenic mice that overexpress AKR1B1 in the lens develop cataracts after induction of galactosemia or experimental diabetes [7]. Therefore, transgenic expression of the human AKR1B10 in the mouse lens allowed us to assess the impact of this enzyme on diabetic cataract formation using a transgenic mouse model that had been validated for diabetic cataract in a previous study. 

In our diabetic animal model studies, we intentionally sought to achieve modest (250–350 mg/dL) levels of hyperglycemia so as to closely mimic the situation experienced by human patients with poorly controlled diabetes. Since the AKR1B10 transgenic mice remained cataract-free throughout 6 months of experimentally induced diabetes, it seems reasonable to conclude that AKR1B10 likely has a limited role in the pathogenesis of diabetic cataract.

## 5. Conclusions

Both AKR1B1 and AKR1B10 are produced in many tissues of the eye affected by diabetes, including cornea, iris, ciliary body, lens, and retina. Because lens transparency was maintained in AKR1B10 transgenic mice following 6 months of experimental diabetes, we conclude that AKR1B10 has a limited role in the pathogenesis of cataract in human patients with diabetes.
